# Effective vaccination strategies to control COVID-19 in Korea: a modeling study

**DOI:** 10.4178/epih.e2023084

**Published:** 2023-09-07

**Authors:** Youngsuk Ko, Kyong Ran Peck, Yae-Jean Kim, Dong-Hyun Kim, Eunok Jung

**Affiliations:** 1 Department of Mathematics, Konkuk University, Seoul, Korea; 2 Division of Infectious Diseases, Department of Medicine, Samsung Medical Center, Sungkyunkwan University School of Medicine, Seoul, Korea; 3 Department of Pediatrics, Samsung Medical Center, Sungkyunkwan University School of Medicine, Seoul, Korea; 4 Department of Social and Preventive Medicine, Hallym University College of Medicine, Chuncheon, Korea

**Keywords:** COVID-19, Models, Theoretical, Public health, Vaccines

## Abstract

**OBJECTIVES:**

In Korea, as immunity levels of the coronavirus disease 2019 (COVID-19) in the population acquired through previous infections and vaccinations have decreased, booster vaccinations have emerged as a necessary measure to control new outbreaks. The objective of this study was to identify the most suitable vaccination strategy for controlling the surge in COVID-19 cases.

**METHODS:**

A mathematical model was developed to concurrently evaluate the immunity levels induced by vaccines and infections. This model was then employed to investigate the potential for future resurgence and the possibility of control through the use of vaccines and antivirals.

**RESULTS:**

As of May 11, 2023, if the current epidemic trend persists without further vaccination efforts, a peak in resurgence is anticipated to occur around mid-October of the same year. Under the most favorable circumstances, the peak number of severely hospitalized patients could be reduced by 43% (n=480) compared to the scenario without vaccine intervention (n=849). Depending on outbreak trends and vaccination strategies, the best timing for vaccination in terms of minimizing this peak varies from May 2023 to August 2023.

**CONCLUSIONS:**

Our findings suggest that if the epidemic persist, the best timing for administering vaccinations would need to be earlier than currently outlined in the Korean plan. It is imperative to continue monitoring outbreak trends, as this is key to determining the best vaccination timing in order to manage potential future surges.

## GRAPHICAL ABSTRACT

[Fig f7-epih-45-e2023084]

## INTRODUCTION

Since the Omicron wave of coronavirus disease 2019 (COVID-19) in early 2022, the Korean government has progressively eased non-pharmaceutical interventions (NPIs). The national social distancing strategy was officially discontinued on April 18 of the same year [1]. Additionally, the mandate to wear masks in outdoor settings was modified to a recommendation on September 26, 2022. The policy on indoor mask-wearing was similarly relaxed on January 1, 2023, apart from public transportation and hospitals. The requirement for mask-wearing on public transportation was later lifted on March 20, 2023 [2-4]. This left the mask mandate for hospitals as the only remaining NPI in effect in Korea. As of May 2023, approximately 20,000 cases are confirmed and 200 severely ill patients are admitted to hospitals in Korea daily [5]. The pursuit of a maximally effective vaccination strategy is crucial, as COVID-19 should ideally be manageable as a respiratory tract infection using medical tools, without the need to reimpose NPIs unless more virulent variants emerge in the future.

Mathematical modeling serves as a valuable instrument for formulating intervention strategies and predictions. As such, numerous models have been developed to offer scientific insights into vaccination strategies. Since the commencement of vaccinations in early 2021, research has been conducted on prioritization based on factors such as age group, region, and risk level. Ko et al. [6] devised a mathematical model that takes into account age group, yielding an age-based vaccine prioritization strategy that varies depending on the outbreak situation. Matrajt et al. [7] proposed that vaccination priority should be given to individuals who are at high risk of severe disease or death. Similarly, Bubar et al. [8] evaluated the effectiveness of various vaccine prioritization strategies, taking serostatus into consideration. Moore et al. [9] determined that the most effective strategy for controlling the spread of COVID-19 was to combine vaccination with NPIs. In the wake of the 2023 Omicron wave, Are et al. [10] discussed how the long-term burden and dynamics of COVID-19 may be influenced by waning immunity and the emergence of new variants, considering various factors. However, their model did not account for residual immunity against disease severity induced by natural infection.

On March 22, 2023, the Korean government transitioned to an annual vaccination schedule and declared that vaccinations would commence between October and November of 2023 [11]. The immunity conferred by vaccines and infections can diminish over time, potentially triggering an epidemic wave [12]. The question of “Who should be vaccinated, and when is the best time?” is a relevant issue, as is the anticipation of future surges. In this paper, we explore effective vaccination strategies for managing COVID-19 surges using a sophisticated mathematical model. Our model, which is based on publicly available data and a national antibody survey, provides a detailed understanding of immunity levels, depending on whether an individual has been vaccinated or infected.

## MATERIALS AND METHODS

### Mathematical modeling of COVID-19 considering immunity induced by vaccine or infection

**Figure 1.**
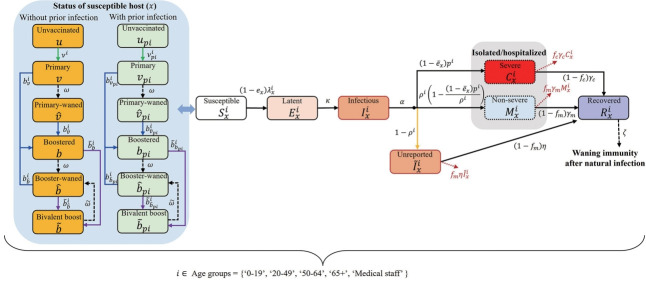
Flow chart for the mathematical modeling of coronavirus disease 2019.

In this study, the population was divided into 5 groups: those aged 0-19 years (*I*), 20-49 years (*II*), 50-64 years (*III*), and 65 years and older (*IV*), as well as medical personnel (*V*). Individuals within these groups were further classified based on their vaccination status. The categories were as follows: unvaccinated, received primary vaccination less than 6 months ago, received primary vaccination 6 or more months ago, received booster vaccination (including third and more doses) less than 6 months ago, received booster vaccination (including the bivariant vaccine) 6 or more months ago, and received the bivariant vaccine less than 6 months ago. Finally, these individuals were additionally categorized based on their prior infection status. Our model, represented as a flowchart in Figure 1, considers population-level immunity, unreported cases, and patients with severe disease. The subscript (*x*) of the classes *S, E, I, C, M, R,* $\tilde{I}$, and *D* indicates the immunity-related status of the host, while the superscript (*i*) denotes the age group.

The transmission matrix for age groups includes various settings such as households, schools, workplaces, hospitals, and other locations [13,14]. This matrix also incorporates statistical data on outpatient and inpatient patients categorized by age group, along with the presumed number of contacts for outpatients (1 each for doctors and nurses) and inpatients (2 for doctors and 3 for nurses). Given that the patterns of contact and transmission in workplaces, schools, and other venues may fluctuate over time, the generalized formula for the elements constituting the transmission matrix is articulated as follows:


$$\lambda_{x}^{i}=\sum_{k\in VG}\sum_{j\in AG}h\delta^{i}\left(c_{1}^{ij}+s\left(t\right)\left(q_{2}c_{2}^{ij}+q_{3}c_{3}^{ij}+g\left(t\right)q_{4}c_{4}^{ij}\right)+q_{5}c_{5}^{ij}\right)\left(I_{k}^{j}+{\tilde{I}}_{k}^{j}\right)$$

where *h* represents the probability of infection through household contact; *δ^i^* denotes the age-dependent relative susceptibility compared to those aged 0-19 years (i.e., *δ^Ⅰ^* = 1) considering clinical susceptibility and other factors, such as compliance with policy or behavior; and *q_2_*, *q_3_*, *q_4_*, and *q_5_* indicate relative risks of infection through contacts at work, other venues, schools, and hospitals, respectively. We assumed that the risk of infection through contact in school was the same as that in households—that is, *q_4_*= 1. However, this was considered to be influenced by the transmission rate adjusting factor *s(t)*, estimated every 4 weeks to fit the data, as well as the school operation factor *g(t)*, deemed to be 0 during vacation periods and 1 otherwise. In other words, contact in school is assumed to be non-existent during vacation periods. The relative risks associated with work-related and miscellaneous contacts—*q_2_* and *q_3_*—were both set to 0.30, while *q_5_* was set to 0.06, reflecting the effectiveness of hospital mask policies [15-17]. The force of infection is reduced by 1−*e_x_* if the susceptible host has been vaccinated and/or infected previously. Similarly, the corresponding severity rate is reduced by 1−${\bar{e}}_{x}$. The unknown parameters *s(t), h*, and *δ^i^* are estimated using the cumulative number of confirmed cases by age. Detailed explanations of the formulae, estimated parameters, and results can be found in the Supplementary Material 1. In this study, the discrepancy between the proportion of confirmed cases and the positive rate of N-antibodies within each age group was incorporated into the model as the rate of unreported cases, 1−*ρ_i_*.

To establish the initial conditions for this mathematical model, we considered groups of individuals with and without previous infections, segmented by vaccination history. This was based on vaccination statistics and the findings of the second Korean national antibody survey, which included the N-antibody positive rate, conducted in mid-December [18]. During this process, we adjusted the population with prior infection to account for the effects of vaccination. Consider a situation in which the vaccine effectiveness is 50%, and for every 100 individuals with prior infection, 100 are vaccinated and 50 unvaccinated. In this case, the number of vaccinated individuals with prior infection is 25 (50× 100× 0.5÷(100× 0.5+50)= 25), while the number of unvaccinated individuals previously infected is also 25 (50× 50÷(100× 0.5+50)= 25). In this study, we assumed that half of the population with previous infections belonged to Ri x and retained full immunity.

### Vaccination time-dependent forecast scenario design

We conducted a simulation of COVID-19 outbreaks in Korea, extending to the situation at the time of this research, for the purpose of parameter estimation. Following this, we extended the simulation for an additional year, starting from May 11, 2023, to determine the best timing for vaccination. To establish the quantity of vaccines needed, we referred to the number of doses administered during the winter vaccination period at the end of 2022, which was approximately 6.6 million doses over 100 days [19]. We then established that 5 million doses of the bivalent vaccine would be administered over the course of 100 days, a figure that is lower than the results from the previous winter vaccination period. We created various scenarios by adjusting the target groups for vaccination, the number of extra doses, and the trends of the outbreak. The vaccination timing was set to begin on the start date of the model simulation extension (May 11, 2023) and to continue for 300 days thereafter (until March 6, 2024), with 10-day intervals. During the extension period, we incorporated the use of antiviral medications, with the default settings assuming these would be prescribed to 40% of patients over the age of 50 years. The application of antiviral drugs resulted in a 51% reduction in the number of severe cases [20].

### Ethics statement

The study was conducted according to the guidelines of the Declaration of Helsinki, and approved by the Institutional Review Board of Konkuk University (7001355-202101-E-130).

## RESULTS

**Figure 2.**
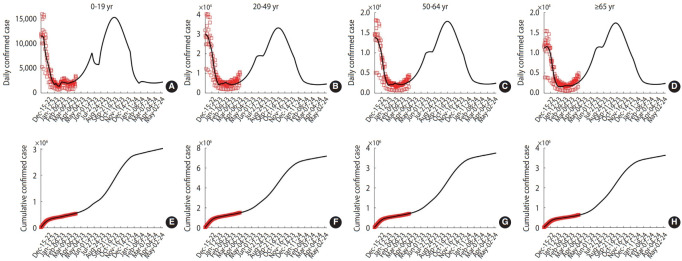
Results of parameter estimation and 1-year extension simulation. (A) through (D) (and (E) through (F)) represent daily confirmed cases (and cumulative confirmed cases) within each age group. The model simulation results are represented by dark curves, while the real-world data are indicated by red squares.

The values and distributions of the estimated parameters can be found in the Supplementary Material 2. The transmission rate adjusting factor s(t) was considered to fluctuate cyclically at 4-week intervals, with values of 0.49, 0.19, 0.82, 0.50, and 0.57. The results of the model simulation, which include data fitting and the 1-year extension, are presented in Figure 2. For the extension, the transmission rate adjusting factor was applied from April 6, 2023 to May 11, 2023, during which time no vaccinations or antiviral medications were administered. The next peak is projected to occur in mid-October 2023. Notably, the sudden decrease observed in the underage group in July can be attributed to the summer vacation period.

**Figure 3.**
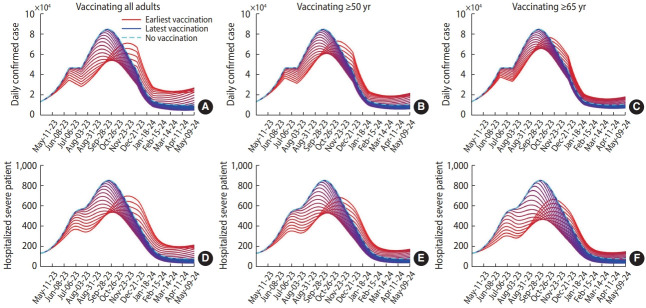
Results of time series extension simulation. (A) to (C) represent daily confirmed cases, while (D) to (F) indicate hospitalizations with severe symptoms. These are shown under different vaccination target settings (represented by each column) and times of vaccination. The dashed cyan curves denote simulation results with no vaccination, while all other curves represent simulation outcomes under a range of vaccination conditions. The curves are presented in a gradient that transitions from red to blue, in ascending order of initial vaccination time.

**Figure 4.**
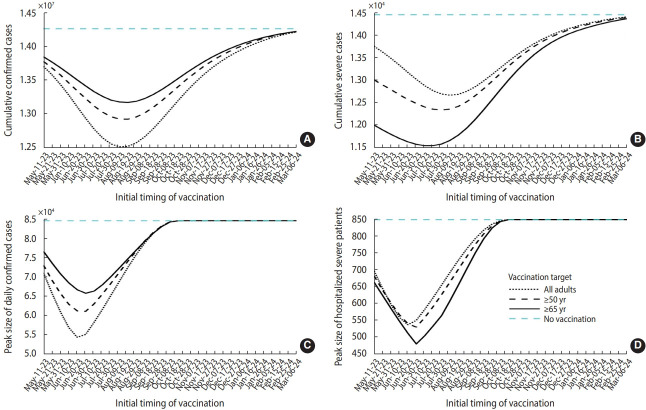
Extended simulation results based on different vaccination targets and initial times of vaccination. (A) and (B) display cumulative confirmed and severe cases, respectively, whereas (C) and (D) represent peaks of daily confirmed cases and severe hospitalizations, respectively. Notably, the x-axis indicates the initial time of vaccination.

Figure 3 presents the simulation results, which reflect various vaccination target settings. The peak of the outbreak can be delayed from mid-October 2023 to mid-November 2023 if a vaccination program is initiated earlier. When all adults were targeted for vaccination, both the daily and cumulative case numbers were lower. However, preferentially vaccinating the elderly resulted in fewer severe cases. Figure 4 summarizes the projected outbreak based on the initial timing of vaccination. If vaccination begins at the end of May, targeting individuals over 65 years of age (at a time when approximately 30,000 daily confirmed cases are expected), the peak number of severe patients requiring hospitalization could be reduced to 480. Table 1 displays the vaccination schedules expected to minimize either the cumulative or peak number of cases (or the number of patients requiring hospitalization due to severe symptoms). It also includes the predicted outbreak outcomes based on different scenario characteristics.

**Figure 5.**
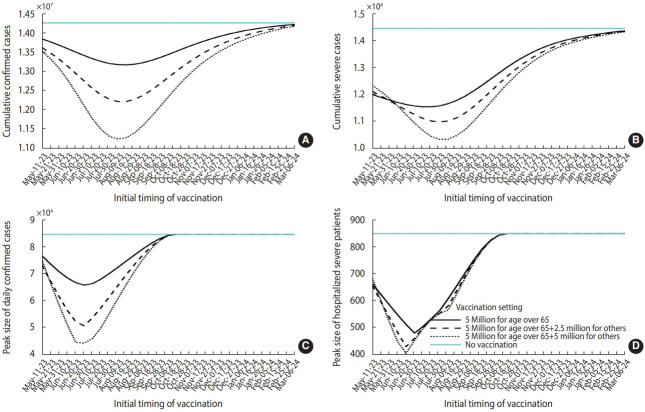
Extended simulation results based on different dosage amounts and initial times of vaccination. (A) and (B) display cumulative confirmed and severe cases, respectively, whereas (C) and (D) represent peaks of daily confirmed cases and severe hospitalizations, respectively. Notably, the x-axis indicates the initial time of vaccination.

Our findings indicated that vaccination strategies targeting individuals over 65 years old minimize the number of severe cases. Consequently, we conducted a supplementary scenario-based study, in which we fixed the number of vaccine doses for the over-65 age group at 5 million and retained the current outbreak trend. However, we varied the additional doses for other adult groups between 2.5 million and 5 million (Figure 5). As the number of extra doses increased, both the cumulative and peak numbers of severe patients were minimized, provided that vaccination commenced at the best time. These additional benefits were diminished if vaccination was initiated earlier or later than the best time point.

When additional analyses were performed for scenarios involving varying numbers of individuals over the age of 65 years receiving vaccinations, the best timing for vaccination differed. Specifically, the best dates were August 9 for 2.5 million individuals and May 31 for 7.5 million individuals, even when assuming the same outbreak trend (Table 1). Supplementary Material 3 provides a description of the sensitivity analysis of fundamental parameters in the extended simulation. This analysis is crucial due to the potential emergence of new variants and future changes in NPIs.

**Figure 6.**
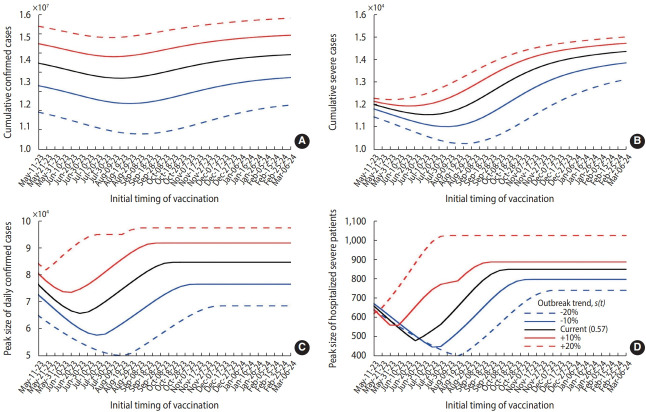
Extended simulation results based on different vaccination outbreak trends and vaccination times. (A) and (B) display cumulative confirmed and severe cases, respectively, whereas (C) and (D) represent peaks of daily confirmed cases and severe hospitalizations, respectively. Notably, the x-axis indicates the initial time of vaccination.

In this study, we set the vaccination count at 5 million for individuals aged over 65 years, as depicted by the solid curves in Figure 6, and extrapolated the simulation results to various outbreak trends, represented by *s(t)* in the model. The best timing to minimize the cumulative or peak numbers of cases and severe hospitalizations may be postponed if *s(t)* decreases. Conversely, if *s(t)* increases, vaccination should commence earlier. For instance, if *s(t)* decreases by 20%, the ideal initiation time for vaccination to minimize the peak number of severe patients is delayed from June 30, 2023 to August 19, 2023.

## DISCUSSION

As the complexity of the mathematical model increases, aggregating the necessary data features becomes increasingly challenging. Moreover, if crucial parameters are assumed or estimated, overfitting could result in erroneous decisions or predictions [21,22]. In this study, we constructed a sophisticated model with parameters and initial conditions that were precisely determined based on data. We took into account hosts who had experienced infection prior to the initial time point, with the Korean antibody survey assisting in establishing the initial conditions of the system ordinary differential equations [18].

As of May 11, 2023, should the current trend continue without further vaccination efforts, the peak of the next wave is predicted to occur in mid-October. The number of severe cases requiring hospitalization could potentially surpass 800. At present, the number of intensive care unit beds designated for COVID-19 patients in Korea stands at 249, a figure that falls short of the anticipated peak demand [23]. Our findings underscore the necessity to adequately equip medical resources in preparation for the forthcoming wave. They also indicate that the impending wave could be handled without resorting to NPIs.

Our simulation results suggest that the best timing of vaccination is earlier than currently outlined in the Korean governmental plan. We discovered that the peak number of severe hospitalizations was minimized when the initial vaccination period fell between May and August, depending on the trend of the outbreak [11]. In the extended simulation results, which were based on the current outbreak trend, we found that the peak was minimized when initial vaccination occurred at the end of June. On the same day as that ideal date of vaccine initiation, the number of daily confirmed cases is projected to reach approximately 30,000, indicating the early phase of a resurgence. However, determining the best timing for vaccination is challenging due to the increasing number of cases, as the outbreak trend is sensitive to changes in key parameters. Therefore, it is crucial to compare future trends with model simulation results to establish effective vaccination scheduling. By considering varying outbreak trends, our model can assist in determining the ideal timing for vaccine administration.

Clearly, an increase in the scale of vaccination leads to a decrease in the scale of the outbreak. However, our observations indicate that hastily implemented large-scale vaccination does not yield a significant effect when compared to a scenario involving lower doses, as shown in panels (B) and (D) of Figure 5. This is because the immunity conferred by vaccination diminishes more rapidly than that induced by natural infection. In a similar vein, we noted that the best timing for vaccination varies in relation to the dosage of the vaccine, as indicated in Table 1, given a consistent outbreak trend. Consequently, the timing of vaccination should be optimized based not only on the outbreak trend, but also on the anticipated scale of vaccination.

The limitations of this study were as follows. First, the contact matrix was established based on data collected prior to the COVID-19 pandemic. Consequently, it may not accurately reflect current conditions due to alterations in population behaviors or lifestyle patterns that have occurred since the outbreak. Second, while the proportion of the population reporting or self-isolating may have changed after December 2022, this information was incorporated into the model using the same values as before. Finally, we did not consider new variants or relaxations in policy that could potentially alter outbreak trends for this prediction. The actual progression of resurgence may deviate from the prediction due to modifications in control strategies for COVID-19 that have been implemented since early June 2023. The results of the sensitivity analysis, found in the Supplementary Material 3, highlight the uncertainty caused by potential changes in key parameters.

## Figures and Tables

**Figure f7-epih-45-e2023084:**
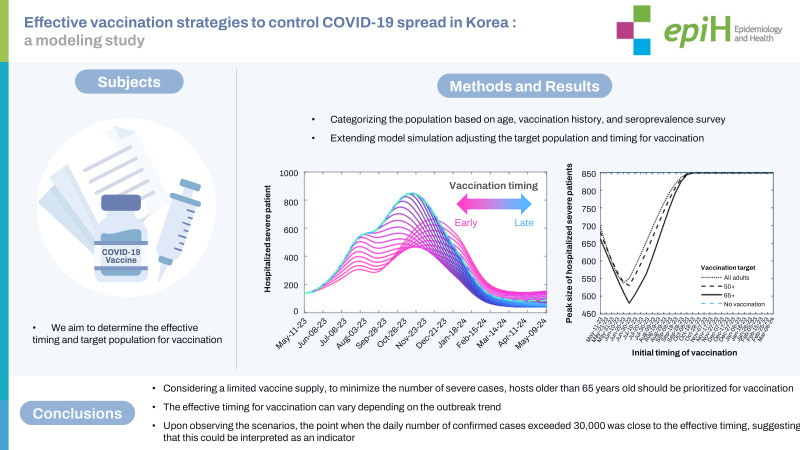


**Table 1.** Best vaccination timing assessments and outbreak predictions according to extended simulation

Variables		No. of doses and vaccination target age (outbreak trend)								
		5 million, all adults (current)	5 million, over 50 yr old (current)	5 million, over 65 yr old (current)	2.5 million, over 65 yr old (current)	7.5 million, over 65 yr old (current)	7.5 million: 5 million over 65 yr old and 2.5 million others (current)	10 million: 5 million over 65 yr old and 5 million others (current)	5 million, over 65 yr old (-20%)	5 million, over 65 yr old (+20%)
Cumulative case minimization										
	Best timing	Aug 9	Aug 9	Aug 19	Sep 8	Jul 30	Aug 9	Aug 9	Sep 8	Aug 9
	No. of cases (millions)	12.52	12.93	13.18	13.66	12.86	12.25	11.26	10.70	14.98
Cumulative severe case minimization										
	Best timing	Aug 9	Jul 30	Jul 10	Aug 9	Jun 20	Jul 30	Jul 30	Aug 29	May 31
	No. of cases (thousands)	12.67	12.35	11.56	12.89	10.47	11.00	10.36	10.27	12.23
Peak daily confirmed cases minimization										
	Best timing	Jun 20	Jun 30	Jun 30	Jul 30	Jun 10	Jun 30	Jun 30	Aug 19	May 21
	Peak case no. (thousands)	54.37	61.12	65.80	72.98	64.20	50.70	42.27	50.21	81.84
Peak administered severe case no. minimization										
	Best timing	20-Jun	30-Jun	30-Jun	09-Aug	May 31	Jun 20	Jun 20	Aug 19	May 11
	Peak case no.	537	530	480	610	420	430	406	401	618

## SUPPLEMENTARY MATERIALS

Supplementary Material 1. System equations of the mathematical model of COVID-19 outbreaks in Korea

Supplementary Material 2 Metropolis-Hasting algorithm application and estimation results

Supplementary Material 3 Sensitivity analysis
