# Supplementary material for: Effective vaccination strategies to control COVID-19 in Korea: a modeling study
Source: Epidemiol Health. 2023 Sep 7;45:e2023084. doi: 10.4178/epih.e2023084 (PMC10867522; doi:10.4178/epih.e2023084)
Supplement: Supplementary Material 2 — Metropolis-Hasting algorithm application and estimation results [file epih-45-e2023084-Supplementary-2.docx]

**Supplementary Material 2**

**Metropolis-Hasting algorithm application and estimation results**

Nine parameters, $h$, $\delta^{Ⅱ}$, $\delta^{Ⅲ}$, $\delta^{Ⅳ}$, $m(t_{1})$, $m(t_{2})$, $m(t_{3})$, $m(t_{4})$, and $m(t_{5})$ are estimated using the Metropolis-Hastings algorithm, which is a Markov chain Monte Carlo method to estimate the distribution of unknown parameters by fitting to the cumulative confirmed cases of the four age groups from December 15, 2022 to May 11, 2023.^[[1]](#endnote-1)^ Note that time $t_{1}$ to $t_{5}$ indicate 28-days length intervals from December 15, 2022 to May 11, 2023. We assumed Gaussian additive noise and uniform prior distribution and sampled parameters as follows:

At iteration $i$ with $x_{i}$,

1. Generate $x^{*}$ from a proposal distribution, $x^{*}\sim q(x_{i})$, $q$ is Gaussian

2. Accept or reject with acceptance rate $\alpha=min \left[ 1,\frac{f\left( x^{*}|Y \right)}{f\left( x_{i}|Y \right)} \right]$

Here, the likelihood function is

$$f\left( x_{i}|Y \right)=\int P\left( Y | x,\sigma_{i} \right)P\left( \sigma_{i} \right)d\sigma_{i}\propto\left\| Y-F\left( x_{i} \right) \right\|^{- \frac{m}{2}},$$

where$F\left( x \right)$ is the model output, $\int\sum_{i} \sum_{x} \rho^{i}\alpha I_{x}^{i} dt$, $Y$ is the data with $m$ points, and $P$ is the prior. Detailed description is in the supporting information of the study of Clarke.^[[2]](#endnote-2)^ **Supplementary Figure 1.** displays sampling results. Median values [95% C.I.] of $h$, $\delta^{Ⅱ}$, $\delta^{Ⅲ}$, $\delta^{Ⅳ}$, $m(t_{1})$, $m(t_{2})$, $m(t_{3})$, $m(t_{4})$, and $m(t_{5})$ are 0·35 [0·34, 0·36], 0·97 [0·95, 1·00], 2·10 [2·05, 2·15], 6·88 [6·80, 6·97], 0·49 [0·46, 0·53], 0·19 [0·14, 0·27], 0·82 [0·66, 0·94], 0·50 [0·34, 0·65], and 0·57 [0·40, 0·79], respectively. Uncertainty of the parameter estimation is provided from the distributions of samples.

**Supplementary Figure 1.** Metropolis-Hasting algorithm application results.

**References**

1. Hastings WK. Monte‐Carlo sampling methods using Markov chains and their applications. Biometrika. 1970;57:97‐109. [↑](#endnote-ref-1)
2. Andrejko KL, Pry JM, Myers JF, Fukui N, DeGuzman JL, Openshaw J, Watt JP, Lewnard JA, Jain S, COVID C, COVID C. Effectiveness of face mask or respirator use in indoor public settings for prevention of SARS-CoV-2 infection—California, February–December 2021. Morbidity and Mortality Weekly Report. 2022;71(6):212. [↑](#endnote-ref-2)
