# Supplementary material for: Effective vaccination strategies to control COVID-19 in Korea: a modeling study
Source: Epidemiol Health. 2023 Sep 7;45:e2023084. doi: 10.4178/epih.e2023084 (PMC10867522; doi:10.4178/epih.e2023084)
Supplement: Supplementary Material 3 — Sensitivity analysis [file epih-45-e2023084-Supplementary-3.docx]

**Supplementary Material 3**

**Sensitivity analysis**

Parameters of a mathematical model affect a model output to different extents. The partial rank correlation coefficient (PRCC) method combined with Latin hypercube sampling (LHS) technique is a reliable sensitivity analysis method when the input parameter and output have nonlinear but monotone relation. Detailed description for the method is in ^[[1]](#endnote-1)^. For the PRCC, we considered possible reduction of vaccine effectiveness against infection ($c_{I}$) and severity ($c_{S}$) to consider possible emergence of new variants. To consider possible change of NPIs and behavior of hosts, transmission rate adjusting factor ($m$), and report rate ($\rho$) are also considered. Two types of model outputs are set for PRCC, cumulative number of infections and severe cases. Simulation time is same as extended simulation, and we set that the vaccination begins from the beginning.

The time dependent PRCC values with respect to the cumulative confirmed and severe cases are displayed in **Supplementary Figure 1**. PRCC values of parameter $c_{I}$, $c_{S}$, $m$, and $\rho$ are ranged as [0·97, 0·98], [-0·02, -0·02], [0·89, 0·93], and [-0·68, -0·60], respectively, when the model output is the cumulative infections. For the cumulative severe cases, PRCC values of parameter $c_{I}$, $c_{S}$, $m$, and $\rho$ are ranged as [0·73, 0·98], [0·75, 1·00], [0·50, 0·93], and [-0·66, -0·22], respectively. Our finding shows that both number of infection and severe cases can be significantly changed if the vaccine effectiveness varies, except vaccine effectiveness against severity to the cumulative number of infections. Still, transmission rate adjusting factor and report rate strongly affect the outbreak.


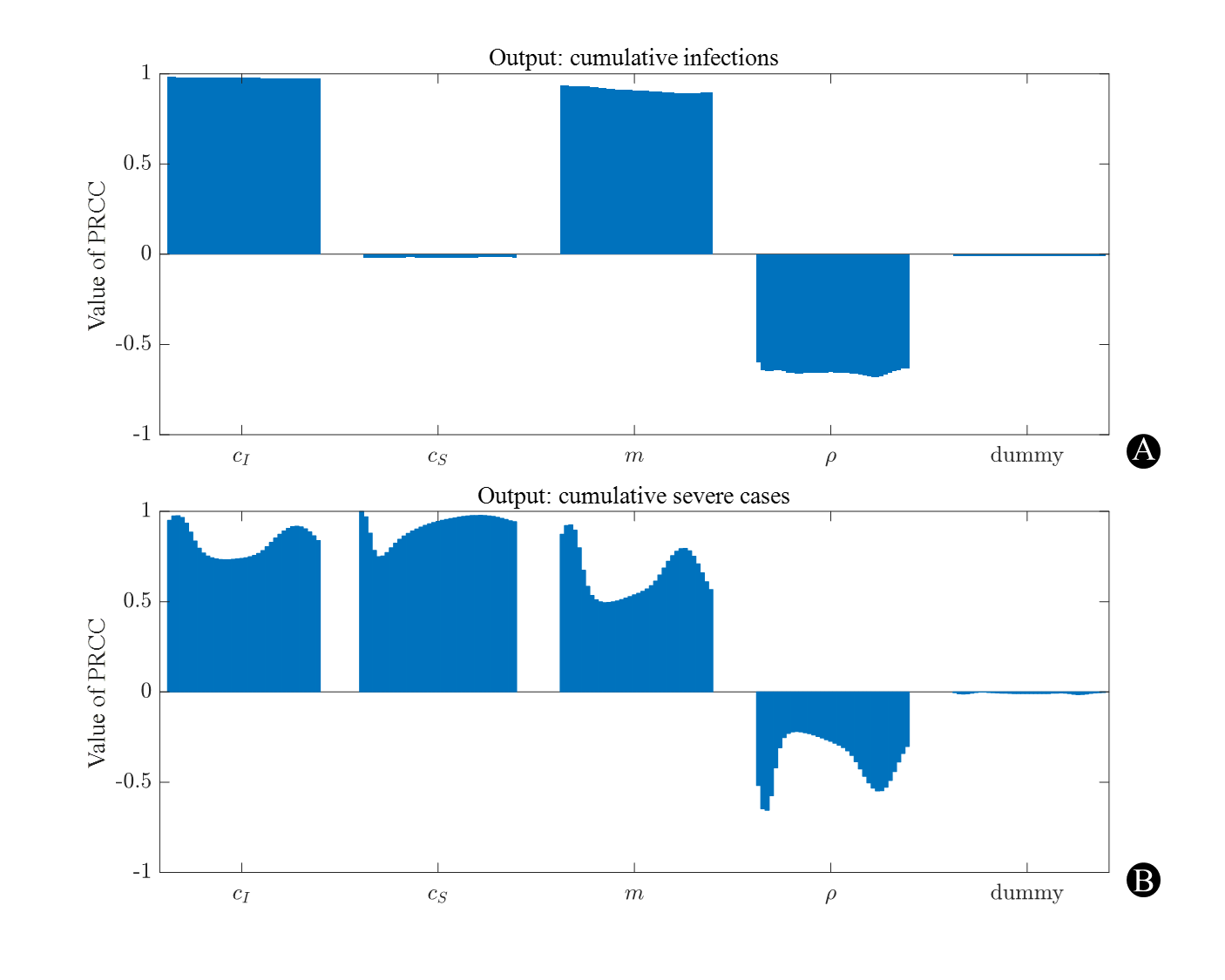
**Supplementary Figure 1.** Time dependent PRCC values of the parameters for model outputs. (A) Output is the cumulative infections (B) Output is cumulative severe cases. Note that dummy parameter does not affect the model at all.

**References**

1. Blower SM, Dowlatabadi H. Sensitivity and uncertainty analysis of complex models of disease transmission: an HIV model, as an example. International Statistical Review/Revue Internationale de Statistique. 1994:229-43 [↑](#endnote-ref-1)
